# Association of Dietary Energy Intake With Constipation Among Men and Women: Results From the National Health and Nutrition Examination Survey

**DOI:** 10.3389/fnut.2022.856138

**Published:** 2022-04-12

**Authors:** Shuai Yang, Xiao-Li Wu, Shou-Qing Wang, Xiang-Ling Guo, Fu-Zheng Guo, Xiao-Feng Sun

**Affiliations:** ^1^Department of Ultrasound, The First Hospital of Jilin University, Changchun, China; ^2^Department of Gastroenterology, Jilin Provincial People's Hospital, Changchun, China

**Keywords:** constipation, energy intake, stool consistency, cross-sectional study, NHANES

## Abstract

**Background:**

Previous studies supported that dietary factor was associated with constipation, but the relationship between dietary energy intake and constipation has not been well-studied. Therefore, we aimed to evaluate the prevalence and correlation between energy intake and constipation among men and women.

**Methods:**

These observational analyses included 12,587 adults (≥20 years) from the 2005–2010 cycles of the National Health and Nutrition Examination Surveys (NHANES). Constipation was defined as Bristol Stool Scale Type 1 (separate hard lumps, like nuts) or Type 2 (sausage-like but lumpy). Total energy intake was obtained from the two 24-h dietary recalls and averaged. We used the logistic regression model in Generalized Linear Model (GLM) function, controlling demographic, lifestyle, and dietary factors, to estimate the association between energy intake and constipation among men and women.

**Results:**

The overall weighted incidence of constipation in this research was 7.4%, the incidence in women and men was 10.4 and 4.3%, respectively. After multivariable adjustment, middle energy consumption correlated with decreased risk of constipation in men (OR:0.5, 95% CI:0.29–0.84), and lower-middle energy intake increased the constipation risk in women (OR: 1.56, 95% CI: 1.15–2.13). High energy consumption was not associated with increased or decreased constipation risk.

**Conclusions:**

To our knowledge, this is the first research to investigate the association between energy intake and constipation; the study demonstrates that appropriate energy consumption can help reduce the risk of constipation in men, and relatively low energy intake is associated with increased constipation risk in women.

## Introduction

Constipation is one of the most common gastrointestinal disorders that heavily affects the quality of life and imparts a tremendous burden on healthcare resources. The estimated prevalence was 10.1% when stricter Rome IV criteria were adopted (1), wherein the incidence of constipation in women had a high reported rate than that of men (17.4 vs. 9.2%), and the risk increased with age growth (2). Based on previous studies, stool consistency described by the validated Bristol Stool Form Scale (BSFS) has been recommended as a validated measure to define constipation compared with stool frequency because BSFS types are better associated with colon transit time (3). Constipation can be generally divided into three types, including normal-transit constipation, slow-transit constipation, and outlet delay disorders. Except for a small portion of patients with structural defects of pelvic floor muscle among them, most individuals with constipation present as functional disturbance of gastrointestinal condition without identifiable structural etiology. Reported general risk factors, such as dietary intake, drug use, lifestyle, the dysfunction of colonic propulsion or rectal emptying, dysbacteriosis, and metabolic disorder, are closely correlated with constipation (4). However, the unsatisfactory treatment outcome raises higher requirements for exploring more complex causes to prevent and improve that condition.

Dietary factors, regarded as modifiable conditions, are generally accepted to be closely linked to the development of chronic constipation. Therefore, in routine clinical practice, dietary management is usually considered the cornerstone of any treatment for chronic constipation. Finite sample size and methods restrict the exploration of complex mechanisms linked to all kinds of foods, so current research mainly focuses on a small part of food ingredients, such as fiber and mineral water (5). Previous studies have confirmed that intake of low liquid, high dietary saturated fat would increase the constipation risk, while intake of soluble fiber, such as Guar gum and Psyllium, and microelements, such as selenium and magnesium, would impose a converse effect (6–9). However, to our knowledge, minimal studies have evaluated the impact of dietary intake on constipation from the perspective of its integrated effects, such as total energy loads, which were mainly supplied by the metabolism of protein, carbohydrate, and fat ingested by us every day. A few pieces of literature have shown that inappropriate intake of macronutrients like a high-fat diet may disturb gastrointestinal motility, suggesting that high energy intake may do as well (10–13). Therefore, we hypothesize that high energy intake may correlate with a higher risk of chronic constipation.

This cross-sectional study aimed to identify the incidence of constipation defined by stool consistency and to investigate the relationship between dietary energy intake and constipation among women and men, with adjustment for other potential confounders, such as other dietary intakes, lifestyle, and demographic factors, by using the National Health and Nutrition Examination Survey (NHANES) database.

## Materials and Methods

### Study Population

National Health and Nutritional Examination Surveys are cross-sectional surveys of a nationally representative sample of the non-institutionalized population conducted by the National Center for health statistics of the Centers for Diseases Control. The stratified and multistage probability cluster design was used in population sampling to estimate the health and nutrition of adults and children in the United States. Written informed consent was provided by all participants. We used the publicly available data from the 2005–2006, 2007–2008, and 2009–2010 NHANES for this study because available bowel health information was provided in these cycles only.

**Figure 1:**
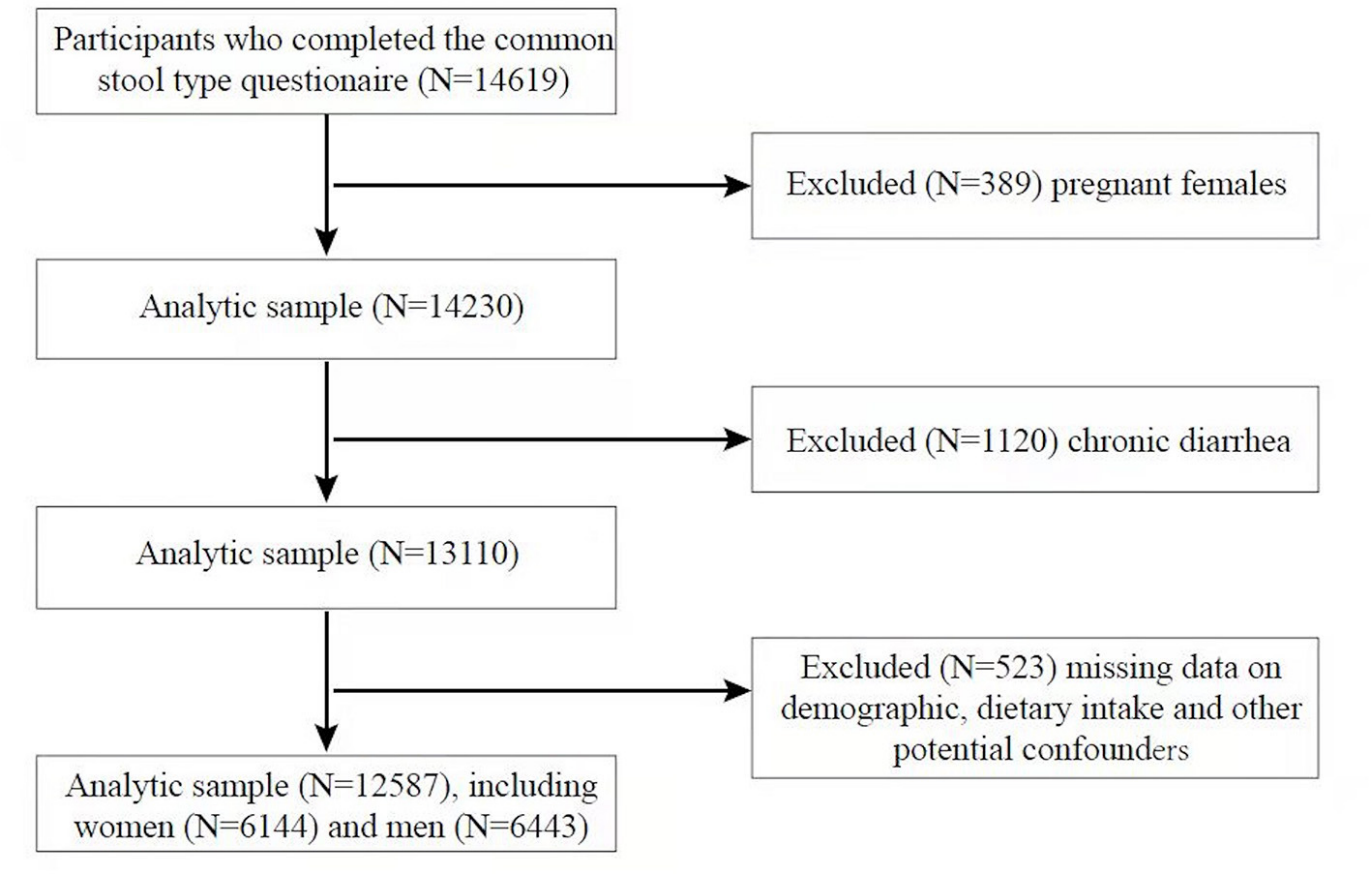
Flowchart of the selection process.

A total of 14,619 men and women aged 20 years or older who completed a common stool type questionnaire were identified. We excluded pregnant women (*N* = 389) and participants with chronic diarrhea (*N* = 1,120) and missing information on demographic data (*N* = 18), dietary intake (*N* = 245), and other potential confounders (diabetes, smoking, drinking, hypertension, vigorous physical activity, *N* = 260), therefore, a sample of 12,587 adults (6,443 men and 6,144 women) was included in our analysis (Figure 1).

### Definition of Constipation

Stool frequency and consistency were recorded in the bowel health questionnaire of the 2005–2010 NHANES, both measures could be used to define constipation. Given the incidence rate of constipation and former studies that estimated constipation in the NHANES surveys, we tend to confirm constipation definition with stool consistency which is assessed by the BSFS. Individuals were shown a card with colored pictures and descriptions of the seven stool types and asked: “Please look at this card and tell me the number that corresponds to your usual or most common stool type.” Constipation was defined as BSFS Type 1 (separate hard lumps, like nuts) or Type 2 (sausage-like but lumpy). Normal stool consistency was defined as BSFS Type 3 (like a sausage but with cracks in the surface), Type 4 (like a sausage or snake, smooth and soft), or Type 5 (soft blobs with clear-cut edges). Chronic diarrhea was defined as BSFS Type 6 (fluffy pieces with ragged edges, a mushy stool) or Type 7 (watery, no solid pieces).

### Dietary Measures

Dietary energy intake among men and women was ascertained by two 24-h dietary recalls. Detailed information about individual foods and amounts of nutrients from each food was provided in that survey. We utilized the average energy intake in the two 24-h recalls or data only from the first 24-h interview if relevant information was missed in the second period. Energy intake was classified as quintiles based on the distribution of intake, men with lower (Q1, 225.1–1,609 kcal/day), lower-middle (Q2, 1,609.1–2,043 kcal/day), middle (Q3, 2,043.1–2464.6 kcal/day), upper-middle (Q4, 2,464.7–3,078.5 kcal/day), and upper (Q5, 3,078.6–13,509 kcal/day) quintiles, and women with lower (Q1, 89–1,215.5kcal/day), lower-middle (Q2, 1,215.6–1,507 kcal/day), middle (Q3, 1,507.1–1,801 kcal/day), upper-middle (Q4, 1,801.1–2,171.7 kcal/day), and upper (Q5, 2,171.8–5,830.5 kcal/day) quintiles.

### Covariates

The age, race/ethnicity, educational level, marital status, smoking, drinking, diabetes, hypertension, body mass index (BMI), vigorous physical activity, and dietary intake, including protein, carbohydrate, fiber, fat, saturated fatty acid, polyunsaturated fatty acid, monounsaturated fatty acid, cholesterol, caffeine, and moisture, were considered as potential confounders in this analysis. Age was classified into groups by decade (20–29, 30–30, 40–49, 50–59, 60–69, 70–79, and ≥80 years old), race/ethnicity was categorized as Mexican American, non-Hispanic white, non-Hispanic black, and other races. Educational level was divided into above high school, high school, below high school. Marital status was grouped into married or living with partner and living alone (widowed, divorced, separated, never married), smoking status was considered as positive when participants have smoked at least 100 cigarettes in their entire life. Drinking status was classified as “yes/no” based on the question: Had at least 12 alcohol drinks/1 year?”. Diabetes status was obtained through the diabetes questionnaire and was dichotomized as “with diabetes,” “without diabetes,“ and ”borderline." Hypertension was considered as positive if participants have ever been told by a health professional that had hypertension; BMI classifications were underweight or normal (BMI <25), overweight (BMI from 25 to 30), and obese (BMI >30). Vigorous physical activity was defined diversely in the cycle of 2005–2006 and 2007–2010. Individuals who answered “yes” in response to “over past 30 days, any vigorous activities for at least 10 min that caused heavy sweating, or large increases in breathing or heart rate in 2005–2006 cycle,” or “vigorous-intensity activity that causes large increases in breathing or heart rate for at least 10 min continuously either at work or during leisure time in 2007–2010 cycle,” were regarded as positive in vigorous physical activity. Dietary intake, such as protein, fiber, caffeine, and moisture, among men and women were obtained from the two 24-h dietary recalls and averaged.

### Statistical Analysis

All data analyses were conducted by the statistical software R (the R Foundation; http://www.r-project.org; version 3.4.3 2021-12-21) and Empower R (www.empowerstats.com; X&Y Solutions, Inc. Boston, MA), which incorporated appropriate sample weights, stratification, and clustering of complex NHANES sampling design. The 6-year weight from dietary interviews was reweighted (1/3 of the 2005–2010 weight) according to the NHANES guidelines. Continuous variables were described by mean, and 95% CIs, and categorical variables were characterized by survey-weighted percentage (95% CI). The survey-weighted Chi-square test tested the difference between categorical variables. The logistic regression model in Generalized Linear Model (GLM) function was adopted to calculate the odds ratios (ORs) and 95% CIs of the correlation between energy intake and constipation in men and women, with the lowest quintile of energy intake as reference. Furthermore, three different regression model was established as diverse confounders were adjusted. Model 1 was adjusted for age, race/ethnicity, educational level, and marital status. Model 2 was adjusted for age, race/ethnicity, educational level, marital status, drinking, smoking, diabetes, hypertension, BMI, and vigorous physical activity. Model 3 was adjusted for covariates in model 2 and dietary intakes of protein, carbohydrate, sugar, fat, polyunsaturated fatty acids, cholesterol, monounsaturated fatty acids, saturated fatty acids, caffeine, and moisture. *P* < 0.05 (two-sided) was thought as statistically significant.

## Results

Table 1 shows that 12,587 adults were included in this analysis, which consisted of 6,443 men and 6,144 women. Among the subjects, the weighted prevalence of constipation among women (10.4%, 688/6,144) was higher than men (4.3%, 331/6,443). Compared with the participants without constipation, men with constipation were more likely to be Mexican-American, have lower educational levels, live alone, have lower BMI, no vigorous physical activity, and a lower intake of protein, fiber, fat, cholesterol, and moisture. Women with constipation tended to be non-Hispanic black, have lower educational levels, have lower BMI, drink, and a lower intake of fiber, fat, monounsaturated fatty acids, Polyunsaturated fatty acids, and moisture in the univariate analysis. Interestingly, although constipation was traditionally considered as being closely associated with age, the incidence of constipation was not increasing with aging as we expected among men and women.

**Table 1:** Participant characteristics by constipation among men and women from National Health and Nutrition Examination Surveys (NHANES) 2005–2010.

**Characteristics**	**Male,** ***N*** **= 6,443**		**^b^** ***P*** **-value**	**Female,** ***N*** **= 6,144**		***^b^P*** **-value**
	**No constipation**	**Constipation**		**No constipation**	**Constipation**	
	**(***N*** = 6,112)**	**(***N*** = 331)**		**(***N*** = 5,456)**	**(***N*** = 688)**	
Age, % (95% CI)^a^			0.007			0.005
20–29	20.3 (18.7, 22.0)	26.0 (19.5, 33.9)		17.0 (15.2, 18.8)	23.1 (19.2, 27.6)	
30–39	19.5 (17.8, 21.4)	15.2 (10.1, 22.1)		17.1 (15.4, 18.9)	14.6 (11.4, 18.5)	
40–49	19.5 (18.2, 20.9)	21.2 (16.8, 26.5)		20.9 (19.4, 22.4)	21.5 (17.8, 25.7)	
50–59	18.8 (17.4, 20.1)	20.8 (14.8, 28.4)		19.9 (18.4, 21.5)	14.3 (10.9, 18.4)	
60–69	12.4 (11.3, 13.6)	5.9 (3.5, 9.7)		13.1 (12.0, 14.3)	14.6 (11.4, 18.5)	
70–79	6.7 (6.0, 7.5)	5.7 (3.6, 8.9)		7.9 (7.0, 8.8)	7.2 (5.7, 9.1)	
>80	2.8 (2.4, 3.3)	5.2 (3.3, 8.2)		4.2 (3.6, 4.9)	4.8 (3.5, 6.4)	
Race/ethnicity, % (95% CI)^a^			<0.001			0.013
Mexican American	8.5 (6.8, 10.7)	16.4 (11.7, 22.6)		6.7 (5.2, 8.6)	7.0 (5.2, 9.4)	
Non-Hispanic white	72.0 (68.3, 75.5)	55.9 (46.2, 65.3)		73.5 (69.6, 77.0)	67.4 (60.2, 73.9)	
Non-Hispanic black	10.2 (8.6, 12.1)	18.2 (13.4, 24.3)		10.8 (8.9, 13.0)	14.5 (10.7, 19.4)	
Others	9.3 (7.8, 11.0)	9.4 (5.1, 16.6)		9.0 (7.5, 10.8)	11.1 (7.6, 16.0)	
Educational level, % (95% CI)^a^			<0.001			<0.001
Below high school	16.8 (15.2, 18.6)	30.7 (23.6, 38.9)		16.4 (14.5, 18.5)	20.8 (17.0, 25.1)	
High school	24.4 (22.5, 26.4)	31.2 (24.5, 38.9)		23.6 (22.0, 25.2)	28.9 (24.3, 34.0)	
Above high school	58.8 (56.0, 61.6)	38.0 (29.2, 47.6)		60.0 (57.5, 62.4)	50.4 (44.6, 56.1)	
Marital status, % (95% CI)^a^			0.02			0.349
Married or living with partner	66.9 (64.9, 68.9)	57.5 (49.3, 65.3)		60.9 (58.1, 63.6)	58.3 (52.8, 63.6)	
Living alone	33.1 (31.1, 35.1)	42.5 (34.7, 50.7)		39.1 (36.4, 41.9)	41.7 (36.4, 47.2)	
BMI(kg/m^2^), %(95% CI)^a^			0.016			0.022
<25 kg/m^2^	26.2 (24.2, 28.4)	35.7 (27.3, 45.1)		36.1 (34.1, 38.1)	39.4 (34.4, 44.6)	
25–30 kg/m^2^	39.4 (37.7, 41.2)	36.9 (30.1, 44.3)		28.4 (26.2, 30.8)	32.7 (28.4, 37.4)	
>30 kg/m^2^	34.3 (32.1, 36.7)	27.4 (22.1, 33.4)		35.5 (33.6, 37.4)	27.9 (23.6, 32.6)	
Drinking status, % (95% CI)^a^			0.100			0.001
Yes	86.1 (84.4, 87.6)	82.1 (77.1, 86.2)		69.1 (66.3, 71.8)	60.8 (55.1, 66.2)	
No	13.9 (12.4, 15.6)	17.9 (13.8, 22.9)		30.9 (28.2, 33.7)	39.2 (33.8, 44.9)	
Smoking status, % (95% CI)^a^			0.465			0.114
Yes	53.3 (51.0, 55.6)	55.9 (48.3, 63.3)		41.8 (39.6, 44.1)	38.0 (33.6, 42.6)	
No	46.7 (44.4,49.0)	44.1 (36.7,51.7)		58.2 (55.9, 60.4)	62.0 (57.4, 66.4)	
Diabetes status, % (95% CI)^a^			0.510			0.043
Yes	7.5 (6.8, 8.4)	6.5 (4.0, 10.3)		7.3 (6.4, 8.3)	9.4 (7.3, 12.1)	
No	90.7 (89.7, 91.5)	92.7 (88.5, 95.4)		91.3 (90.1, 92.3)	89.9 (87.3, 92.0)	
Borderline	1.8 (1.4, 2.3)	0.8 (0.1, 4.5)		1.5 (1.1, 1.8)	0.7 (0.3, 1.7)	
Hypertension, % (95% CI)^a^			0.201			0.565
Yes	29.0 (27.0, 31.1)	24.9 (19.0, 31.9)		31.5 (29.7, 33.4)	30.1 (25.5, 35.2)	
No	71.0 (68.9, 73.0)	75.1 (68.1, 81.0)		68.5 (66.6, 70.3)	69.9 (64.8, 74.5)	
Vigorous physical activity, % (95% CI)^a^			0.037			0.363
Yes	48.8 (46.4, 51.2)	39.8 (32.0, 48.1)		30.9 (28.9, 33.0)	27.9 (22.0, 34.5)	
No	51.2 (48.8, 53.6)	60.2 (51.9, 68.0)		69.1 (67.0, 71.1)	72.1 (65.5, 78.0)	
Quintile energy intake			0.008			0.920
Lower quintile	15.3 (14.1, 16.5)	23.0 (16.9, 30.4)		16.8 (15.4, 18.3)	15.8 (12.8, 19.4)	
Lower-middle quintile	18.6 (17.4, 19.9)	21.6 (15.8, 28.8)		18.9 (17.2, 20.8)	24.1 (19.9, 28.8)	
Middle quintile	20.7 (19.4, 22.0)	12.5 (8.3, 18.4)		21.2 (19.6, 22.8)	21.8 (17.9, 26.4)	
Upper-middle quintile	21.8 (20.4, 23.2)	24.2 (18.4, 31.1)		21.9 (20.4, 23.5)	19.5 (15.6, 24.1)	
Upper quintile	23.7 (22.0, 25.5)	18.8 (13.0, 26.5)		21.2 (19.6, 22.9)	18.7 (15.8, 22.1)	
Protein intake (gm/d)	88.6(87.3, 89.8)	81.4 (76.7, 86.0)	0.006	75.5 (74.5, 76.5)	74.6 (71.0, 78.1)	0.612
Carbohydrate intake (gm/d)	273.3 (269.8, 276.8)	265.1 (249.5, 280.6)	0.313	236.9 (233.4, 240.3)	237.6 (231.3, 244.0)	0.837
Fiber intake (gm/d)	17.2 (16.9, 17.5)	16.0 (14.9, 17.1)	0.026	15.9 (15.7, 16.2)	15.1 (14.5, 15.7)	0.014
Sugar intake (gm/d)	122.7 (120.5, 124.9)	119.1 (108.9, 129.3)	0.483	108.0 (105.9, 110.2)	109.9 (105.2, 114.5)	0.497
Fat intake (gm/d)	85.0 (83.6, 86.4)	80.2 (75.8, 84.5)	0.046	72.7 (71.6, 73.9)	68.9 (66.2, 71.5)	0.019
Saturated fatty acids intake (gm/d)	28.0 (27.5, 28.5)	26.0 (24.3, 27.7)	0.039	23.9 (23.5, 24.3)	22.8 (21.7, 23.9)	0.092
Monounsaturated fatty acids intake (gm/d)	31.2 (30.6, 31.7)	29.8 (28.1, 31.5)	0.149	26.5 (26.1, 26.9)	25.1 (24.1, 26.1)	0.024
Polyunsaturated fatty acids intake (gm/d)	18.3 (17.9, 18.6)	17.3 (16.3, 18.2)	0.057	15.9 (15.6, 16.3)	14.9 (14.4, 15.4)	0.003
Cholesterol intake (mg/d)	318.1 (311.5, 324.6)	281.8 (259.5, 304.2)	0.003	262.3 (256.7, 267.9)	258.2 (242.4, 273.9)	0.598
Caffeine intake (mg/d)	171.1 (165.6, 176.7)	164.5 (136.1, 193.0)	0.664	156.8 (151.1, 162.5)	142.4 (127.3, 157.6	0.064
Moisture intake (gm/d)	3,002.6	2,817.0	0.005	2,758.1	2583.7	0.001
	(2,961.5, 3,043.7)	(2,689.7, 2,944.2)		(2,716.0, 2,800.2)	(2,487.6, 2,679.8)	

a *Survey-weighted percentage (95% CI)*;

b *P-value: survey-weighted Chi-square test*.

Table 2 presents the weighted ORs (95% CI) of constipation based on quintiles of total energy intake. The lower middle dietary intake of energy (1,215.6–1,507 kcal/day) remained correlated with increased constipation risk after covariates adjustment in different models among women. The ORs (95% CI) of constipation for that energy intake level, compared with the lowest quintile, was 1.46 (1.1–1.95) in model 1 (adjusted for age, educational level, race/ethnicity, and marital status), 1.49 (1.11–1.99) in model 2 (adjusted for variables in model 1, and also drinking, smoking, diabetes, hypertension, BMI, and vigorous physical activity), and 1.56 (1.15–2.13) in model 3 (adjusted for covariates in model 2, and also dietary intakes, such as protein and carbohydrate). Other quintiles of energy intake did not show a familiar association with constipation. Among men, the inverse association between middle dietary intake of energy (2,043.1–2,464.6 kcal/day) and constipation (*P* < 0.05) was detected in all models. The ORs (95% CI) of constipation for that quintile was 0.48 (0.27–0.83) in model 1,0.47 (0.27–0.82) in model 20.5 (0.29–0.84) in model 3. As for upper intake of energy (3,078.6–13,509 kcal/day) showed an association with decreased constipation risk in model 1 and model 2 with ORs 0.58 and 0.57, respectively. However, after adjustment for dietary intakes, such as protein, fiber, sugar, cholesterol, caffeine, and moisture, the upper intake of energy was borderline associated with constipation (OR:0.63, 95% CI:0.4–0.99, *p* = 0.065). The association between other quintiles of energy intake and constipation was not seen.

**Table 2:** Weighted ORs and 95% CIs for constipation among men and women according to the quintiles of dietary energy intake.

	**Model 1 OR(95%CI)**	**Model 2 OR (95%CI)**	**Model 3 OR (95%CI)**
**Women**
Q1 (89.0–1,215.5 kcal/day)	1.00 (ref.)	1.00(ref.)	1.00 (ref.)
Q2 (1,215.6–1,507.0 kcal/day)	1.46 (1.10–1.95)*	1.49 (1.11–1.99)*	1.56 (1.15–2.13)*
Q3 (1,507.1–1,801.0 kcal/day)	1.19 (0.85–1.65)	1.21 (0.86–1.69)	1.25 (0.88–1.78)
Q4 (1,801.1–2,171.7 kcal/day)	1.05 (0.72–1.51)	1.07 (0.74–1.55)	1.14 (0.75–1.72)
Q5 (2,171.8–5,830.5 kcal/day)	0.99 (0.73–1.33)	1.05 (0.77–1.44)	1.13 (0.77–1.65)
**Men**			
Q1 (225.1–1,609.0 kcal/day)	1.00 (ref.)	1.00 (ref.)	1.00 (ref.)
Q2 (1,609.1–2,043.0 kcal/day)	0.86 (0.53–1.38)	0.85 (0.53–1.37)	0.90 (0.57–1.41)
Q3 (2,043.1–2,464.6 kcal/day)	0.48 (0.27–0.83)*	0.47 (0.27–0.82)*	0.50 (0.29–0.84)*
Q4 (2,464.7–3,078.5 kcal/day)	0.86 (0.58–1.27)	0.86 (0.59–1.27)	0.94 (0.63–1.39)
Q5 (3,078.6–13,509.0 kcal/day)	0.58 (0.35–0.94)*	0.57 (0.34–0.94)*	0.63 (0.40–0.99)

*OR, odds ratio; CI, confidence interval. Model 1 adjusted for age, race/ethnicity, educational level, and marital status; Model 2 adjusted for age, race/ethnicity, educational level, marital status, drinking, smoking, diabetes, hypertension, BMI, and vigorous physical activity; Model 3 adjusted for age, race/ethnicity, educational level, marital status, drinking, smoking, diabetes, hypertension, BMI, vigorous physical activity, and dietary intake of protein, carbohydrate, sugar, fat, saturated fatty acids, monounsaturated fatty acids, polyunsaturated fatty acids, cholesterol, caffeine, and moisture*.

* *P < 0.05*.

## Discussion

We estimate the correlation between dietary energy intake and chronic constipation with a large nationally representative adult sample for the first time. Patients with constipation often display a range of symptoms, including hard stools, infrequent bowel movements, excessive straining, and abdominal pain, and often defined by self-reported symptoms, stool frequency, and the Rome Criteria (14). From the perspective of clinical implication, using the stool consistency defined by BSFS to diagnose constipation has been more readily accepted. In this study, the overall weighted incidence rate of constipation defined by stool consistency is 7.4%, women have a higher incidence rate of 10.4% than men of 4.3%. The latest authoritative data from a meta-analysis shows that the global prevalence of functional constipation is at least 10.1% with Rome criteria (1), and incidence of constipation is higher in women, using stool-consistency-based definition does underestimate the prevalence of constipation, but accord with risk distribution between women and men.

These observations reveal an inverse effect of dietary energy intake on constipation between men and women in the general adult population for the first time. Compared with the lowest quintile, a relatively low intake of energy (1,215.6–1,507 kcal/day) correlates with a higher risk of constipation in women, an inverse correlation between a middle intake of energy (2,043.1–2,464.6 kcal/day) and constipation risk is observed in men, even after adjustment of other confounders for constipation, including age, race/ethnicity, educational level, marital status, drinking, diabetes status, hypertension, BMI, vigorous physical activity, and dietary intake, such as protein and moisture. As for other quintiles of energy intake, no statistical significance was detected in women and men. A smaller study in children has shown that symptoms of constipation were relieved with an increased intake of daily energy during treatment, suggesting that adequate energy intake may correlate with decreased constipation risk (15). According to the eighth edition of dietary guidelines for Americans, recommended energy intake every day ranges from 1,600 to 2,400 kcal for adult women and 2,000 to 3,000 kcal for adult men (16). For women, energy intake, which enhances constipation risk, is below the lower limit of the daily dietary allowance of energy. This result may provide guiding significance for weight-watcher, in which inappropriate reduced energy intake for weight loss may contribute to other problems risk like constipation. Within the range of recommended energy intake, the men can reduce the risk of functional constipation.

There are several potential explanations for the observed dissimilar correlation between energy intake and constipation among women and men. Generally, in healthy adults, food intake can acutely increase the colon's motility to cause the desire to defecate, which is triggered by dilatation of the stomach and catabolite of the small intestine; this phenomenon is also called gastrocolic reflex (17). For patients with slow transit constipation, intake of energy cannot mediate the normal increase of the distal colonic cyclic propagating motor patterns, and defecation desire fails to be activated (18). Our study results show that lower energy consumption may cause a decrease in gastrointestinal (GI) motility and increased constipation in women; middle energy intake can improve GI motility and reduce the risk of constipation in men. Luscombe-Marsh Natalie et al. suggest that energy density intake increased to a certain extent would delay gastric emptying, and more subsequent energy intake will not accelerate gastric emptying either (19). We believe that different levels of energy intake might also exert a differential effect on gastrointestinal motility and constipation. Wilbrink et al. show that calorie loads might act as an essential factor in mediating the ileal brake effect on gastric emptying, and intestinal transit irrespective of types of macronutrients intake (20). We speculate that men's appropriate energy intake (2,043.1–2,464.6 kcal/day) may successfully activate the gastrocolic reflex of patients with normal colon transit; ileal brake cannot be induced under that degree of calorie loads, and then higher energy intake may result in a reduction of gut transit in men. Nevertheless, for women, relatively low-calorie loads (1,215.6–1,507 kcal/day) may activate the small intestine brake effect, hence slowing gastrointestinal transit and predisposing to constipation. The potential mechanism of calorie load affecting gastrointestinal motility and risk diversity between women and men still needs further investigation.

The present study has several strengths. First, to our knowledge, this study is the first to assess the relationship between energy intake and constipation among men and women using the largest nationally representative sample. Second, it is also the first study to show the inverse effect of energy intake with constipation between men and women. Third, we adjusted for the possible confounders to obtain more reliable results. Nevertheless, several limitations exist in this study. First, this is a cross-sectional study, and we cannot determine the causation and the temporal relationship between the onset of constipation and energy intake. Second, we cannot use the standard of the Rome criteria to define constipation more accurately, and classification of constipation is not mentioned in the NHANES database; the prevalence of constipation might be underestimated with BSFS to define constipation. Third, the NHANES database is based on self-report; the recall bias may exist when investigating the most common stool type and two 24-h dietary intake recalls. Fourth, although we have adjusted for main potential confounders, other unobserved factors may still exist.

## Conclusions

In summary, we estimate the association between dietary energy intake and constipation with the nationally-representative sample for the first time. The present study indicates that middle energy consumption (within recommended energy intake) can help reduce the risk of constipation in men, and relatively low energy intake (below recommended energy intake), correlates with increased constipation risk in women. We believe that discovery provides new insight for constipation management from the perspective of total energy loads. However, more research is needed to support appropriate energy intake can ameliorate constipation.

## Data Availability Statement

The raw data supporting the conclusions of this article will be made available by the authors, without undue reservation.

## Ethics Statement

The studies involving human participants were reviewed and approved by the Ethics Review Board of Centers for Disease Control and Prevention (CDC). The patients/participants provided their written informed consent to participate in this study.

## Author Contributions

X-FS and SY designed the study. S-QW and X-LG acquired the data. X-LW and F-ZG analyzed the data. SY drafted the manuscript. X-FS and X-LW critically revised the manuscript. All authors have read and approved the final manuscript.

## Conflict of Interest

The authors declare that the research was conducted in the absence of any commercial or financial relationships that could be construed as a potential conflict of interest.

## Publisher's Note

All claims expressed in this article are solely those of the authors and do not necessarily represent those of their affiliated organizations, or those of the publisher, the editors and the reviewers. Any product that may be evaluated in this article, or claim that may be made by its manufacturer, is not guaranteed or endorsed by the publisher.
